# Sulfonated Poly(ether ether ketone)–Zirconia Organic–Inorganic Hybrid Membranes with Enhanced Ion Selectivity and Hydrophilicity for Vanadium Redox Flow Batteries

**DOI:** 10.3390/polym17172287

**Published:** 2025-08-23

**Authors:** Xiang Li, Tengling Ye, Wenfei Liu, Ge Meng, Wenxin Guo, Sergey A. Grigoriev, Dongqing He, Chuanyu Sun

**Affiliations:** 1 Department of Applied Chemistry, School of Chemistry and Chemical Engineering, Harbin Institute of Technology, Harbin 150001, China; 2 Yantai Research Institute, Harbin Engineering University, Yantai 264003, China; 3 School of Electrical Engineering and Automation, Harbin Institute of Technology, Harbin 150001, China; 4 Department of Proving Ground, FAW-Volkswagen Automotive Company Limited, Changchun 130011, China; 5 National Research University “Moscow Power Engineering Institute”, 14, Krasnokazarmennaya St., 111250 Moscow, Russia; 6 National Research Centre “Kurchatov Institute”, 1, Akademika Kurchatova Sq., 123182 Moscow, Russia; 7 Institute of Advanced Technology, Heilongjiang Academy of Sciences, Harbin 150020, China; 8 Suzhou Research Institute, Harbin Institute of Technology, Suzhou 215104, China

**Keywords:** poly(ether ether ketone), SPEEK, proton-exchange membrane, PEM, flow battery, vanadium flow battery, energy storage system, inorganic–organic hybrid membrane

## Abstract

Proton-exchange membranes (PEMs) are the pivotal components of vanadium redox flow batteries (VRFBs) and play a critical role in the comprehensive output performance of VRFB systems. Currently, the most widely commercialized membranes are Nafion series membranes produced by DuPont, Wilmington, DE, USA, but the high vanadium permeability and cost hinder their large-scale promotion. Hence, there is an active demand for developing a low-cost, high-performance, and energy-efficient PEM to promote the commercialization of VRFB systems. In this paper, sulfonated poly(ether ether ketone) (SPEEK) as matrix and zirconia nanoparticles as inorganic filler were used for composite modification to prepare a series of SPEEK–ZrO_2_ organic–inorganic hybrid membranes for VRFBs. The thickness of these membranes was 50–100 μm. Compared with Nafion 115 (thickness 128 μm), composite membranes demonstrated obvious cost advantages. The results showed that the SP–Z-X series membranes had higher water uptake (53.26–71.1%) and proton conductivity (0.11–0.24 S cm^−1^). SP–Z-5 displayed the best comprehensive output performance at 200 mA cm^−2^ (CE: 99.01%, VE: 81.95%, EE: 81.11%). These hybrid membranes are very cost-effective and exhibit high potential for application in VRFB applications, and are expected to lead to the industrial application of VRFBs on a large scale.

## 1. Introduction

Energy is indispensable for human survival and social development. With the ongoing development of the global economy and increasing population, energy consumption is also rising, resulting in the large-scale use and exploitation of traditional fossil fuels such as natural gas, oil, and coal [1]. While non-renewable energy is facing the issue of energy depletion, it has also caused severe environmental pollution, seriously endangering human health and affecting the environment. To tackle the problem of environmental pollution and the coming shortage of unrenewable energy, many energy suppliers are focusing on renewable energy sources: for instance, tidal energy, hydropower, solar energy, wind energy, and biomass energy. However, these energy sources are intermittent and volatile, and large-scale grid integration will cause profound damage to the power system. Therefore, developing large-scale long-term energy storage systems, including vanadium redox flow batteries (VRFBs) and proton-exchange membrane fuel cells (PEMFCs), to store and release energy for long periods, realizing peak shaving, and thus promoting the utilization of renewable energy is a pressing matter [2,3,4,5]. VRFBs are considered the most suitable candidate for megawatt-level energy storage, and systems producing such levels of power have been operating in China, Australia, and the USA. They are appropriate for the energy storage of photovoltaics and wind power, as well as being connected with the power grid [6,7].

Among the numerous energy storage technologies, VRFBs have broad development prospects due to their low cost, long service life, high efficiency, intrinsic safety, flexible design, and capacity for 100% discharge without damaging the battery. They have become one of the primary battery candidates to fulfill the energy storage demands of future smart grids. The most unique features of VRFBs are their feasibility of use for energy needs and independent scaling up of capacity. The capacity of VRFBs can be enhanced by simply expanding the concentration of electroactive species or volume of the electrolytes, which represents an advantage over other electrochemical energy storage techniques [8,9,10]. All-vanadium RFBs (VRFBs), invented by Maria Skyllas-Kazacos from the University of New South Wales (Australia), are regarded as the most mature RFB system at present. The most significant advantage of this system is that the positive and negative redox couples employed are identical vanadium elements [11,12,13,14]. Due to the four valence states of vanadium ions, the system can effectively avoid performance failure caused by ion crossover. Additionally, the vanadium electrolyte is relatively easy to transfer and store, which greatly reduces the safety hazards during operation [15,16]. In summary, VRFBs are considered one of the most promising RFBs.

Proton-exchange membranes (PEMs) are the core components in these battery systems. The PEM is sandwiched between the two electrodes to separate the electrolytes and prevent the penetration of the catholytes and anolytes. This provides a pathway for ion conduction between the two electrolytes during charging and discharging, and is one of the most vital components of VRFBs. Achieving high ion selectivity, excellent chemical stability, and high proton conductivity of the PEM is crucial to enhance VRFB output performance [17,18,19,20].

The most commercialized PEM currently is the Nafion membrane produced by DuPont in the United States. Nafion is a perfluorosulfonic acid (PFSA) membrane with a hydrophobic polytetrafluoroethylene-like main chain and a hydrophilic perfluorovinyl ether side chain terminated with sulfonic acid groups [21,22,23]. Nevertheless, as a cation-exchange membrane, it also possesses high vanadium species crossover due to the significant phase separation between the hydrophilic side chains and hydrophobic main chains. Hence, the self-discharge phenomenon appears to be due to the vanadium crossover and leads to continuous attenuation in the capacity of the VRFB. Moreover, the extremely high expense of Nafion limits its widespread applications in VRFBs to a certain extent.

Many studies have been dedicated to finding a novel membrane candidate with higher ion selectivity, proton conductivity, and lower cost than Nafion [24,25]. Typically, scholars tend to modify the membrane by polymer blending and introducing inorganic fillers to obtain an inorganic–organic hybrid membrane with better performance [26,27,28].

Tao Luo [29] et al. prepared a porous polybenzimidazole membrane using a phase inversion approach. Compared with Nafion 112, the porous membrane had better chemical stability, lower vanadium species crossover, and realized a coulombic efficiency (CE) of 98%. Zhensheng Mai [30] et al. blended Nafion with polyvinylidene fluoride (PVDF) to prepare a composite membrane for VRFBs. The results demonstrated that the introduction of the hydrophobic polymer PVDF significantly limited the swelling ratio of the membranes, and the composite membrane containing 20 wt% PVDF achieved an energy efficiency (EE) of 85% at 80 mA cm^−2^, which was higher than Nafion. The decay time in open-circuit voltage (OCV) was significantly longer than that of the Nafion 112, which was doubled. Hyeon Jin Choi [31] et al. synthesized a 50 μm Nafion–MOF hybrid membrane by incorporating an Al-based nanoporous metal–organic framework in a Nafion matrix, which had a significant cost advantage compared to Nafion 115 (127 μm). The test results revealed that this composite membrane exhibited better battery performance than the Nafion membrane. A composite membrane containing 6 wt% MOF can achieve an EE of 77.56% at 160 mA cm^−2^. After 100 charging–discharging cycles of a VRFB single cell, the VRFB with a composite membrane had a capacity retention rate of 80.34%. Y.H. Wan [24] et al. prepared Nafion nanofibers by electrospinning and embedded them in PBI to prepare composite membranes. The nanofiber structure was able to serve as a continuous proton conduction pathway, achieving high proton conductivity of the blended membrane under the conditions of low Nafion content. Moreover, the acid–base interaction between Nafion and PBI significantly improved the mechanical stability of the composite membrane. Compared with Nafion 212, the blended Nafion–PBI membrane with 40 wt% Nafion exhibited an excellent vanadium ion-blocking effect that was 58 times that of the pristine Nafion membrane. In the VRFB single-cell test, the composite membrane achieved 99.8% CE and 80% EE at up to 210 mA cm^−2^.

In addition, non-fluorinated polymer, materials including sulfonated poly(ether ether ketone) (SPEEK) [32,33], sulfonated polyether sulfone (SPES) [34,35,36], sulfonated polyimide (SPI) [37,38,39], and imidazole polymer (PBI) [29,40] have gradually become research hotspots in recent years. In terms of VRFB application, these non-fluorinated PEMs have exhibited significant advantages over PFSA membranes in many aspects. The raw materials used to prepare non-fluorinated PEMs are mostly common polymer materials, which are inexpensive and widely available, significantly reducing the raw material cost of the membrane. Furthermore, preparing them is relatively simple, typically completed using conventional methods such as solution casting and melt blending, without the need for complex steps such as the polymerization of fluorinated monomers. For example, when preparing a polyvinyl chloride and polyvinyl pyrrolidone composite proton-exchange membrane, simply adjusting the ratio of PVP and PVC in the composite membrane can produce a PEM with performance suitable for VRFBs. This simple process not only reduces the investment in production equipment but also simplifies process control, further reducing the overall production cost. In contrast, PFSA membranes rely on expensive fluorine-containing raw materials, and their complex synthesis process results in high prices. This has become a significant cost constraint in the application of large-scale VRFBs as energy storage systems [41,42]. Moreover, the production process of non-fluorinated PEMs does not involve the use or emission of fluorinated compounds, making them environmentally friendly. However, byproducts such as perfluorooctanoic acid (PFOA) produced in the production of PFSA membranes are persistent organic pollutants that do not degrade easily in the natural environment and can accumulate in water bodies, soil, and other pathways over long periods of time, posing a potential threat to the ecological environment and human health. Throughout their entire life cycle, from production to disposal, non-fluorinated PEMs have less negative impact on the environment, are more in line with the requirements of green and sustainable development, and are particularly suitable for energy storage projects with strict environmental standards [43].

PEEK is a kind of engineering plastic with excellent oxidative stability, thermal stability, and mechanical properties, and SPEEK is considered one of the most promising membrane materials to replace Nafion. The SPEEK main chain is composed of rigid benzene rings with side chains that contain hydrophilic sulfonic acid groups, and possesses a chemical structure similar to that of perfluorosulfonic acid membranes.

Researchers have investigated the performance of VRFBs containing SPEEK membranes with various degrees of sulfonation (DS) and multiple casting solvents. Jingyu Xi et al. revealed that SPEEK (DS = 67%) and DMF as the casting solvent demonstrated optimal VRFB performance (an EE of 85% at 80 mA cm^−2^) in terms of electrochemical performance [44]. However, as DS increases, SPEEK exhibits greater water uptake and swelling ratio due to the increased number of hydrophilic -SO_3_H groups. Increased hydrophilicity leads to higher proton conductivity, while excessive swelling significantly reduces mechanical properties. When DS increases from 57% to 87%, the proton conductivity of SPEEK membranes increases from 4.2 mS cm^−1^ to 13.4 mS cm^−1^, but its fracture strength decreases from 49.9 MPa to 24.1 MPa.

Bibo Yin et al. synthesized three families of SPEEK hybrid membranes by incorporating three inorganic nanoparticles of Al_2_O_3_, TiO_2_, and SiO_2_ into SPEEK, which significantly enhanced the chemical stability, mechanical stability, and thermal stability of the PEM materials [45]. During VRFB operation, the EEs of the VRFBs containing hybrid membranes performed better than Nafion 117.

Many researchers are also committed to improving the thermal stability, chemical durability, and mechanical properties of SPEEK membranes to promote their widespread application in fuel cells. In recent years, significant progress has been made in modifying SPEEK for fuel cell applications. Ae Rhan Kim et al. embedded amine-functionalized carbon nanotubes (ACNTs) as inorganic potential proton-conducting materials into the SPEEK matrix [46]. The morphology, structure, thermomechanical, physicochemical, and electrochemical properties of the SPEEK–ACNT composites were investigated and compared with those of pristine SPEEK. The results showed that the synthesized composite membranes exhibited uniform and dense morphology and enhanced the performance of PEMFCs at low relative humidity. SPEEK–ACNT (1.5 wt%) exhibited improved PEMFC performance at 20% RH and exhibited superior durability compared to SPEEK. To improve the durability and performance of SPEEK, Somayeh Sarirchi et al. prepared a series of hybrid membranes by doping sulfated titania and sulfated zirconia–titania into the SPEEK matrix [47]. Structural, morphological, thermochemical, and mechanical tests were performed on the membrane. The results showed that the performance of the nanocomposite membrane was significantly improved compared to pristine SPEEK. An MEA based on the nanocomposite membrane achieved a peak power density of 500 mW cm^−2^ at a current density of 794.7 mA cm^−2^ at 120 °C and RH = 80%.

In this study, a novel family of SPEEK–ZrO_2_ hybrid membranes with various ZrO_2_ content was prepared and specifically applied to an all-vanadium redox flow battery system. In order to further improve the properties of the membrane materials while ensuring good proton conductivity, a SPEEK with a DS of 74% was used. By conducting targeted performance optimization and verification centered on the core needs of this scenario, we found that the modified membranes performed better than VRFBs. Due to the good hydrophilicity and high specific surface area of zirconium oxide particles, they enhanced the proton conductivity and water uptake of the hybrid membrane to a certain extent. The vanadium ion permeability of the hybrid membrane was 41% lower than that of the pristine SPEEK membrane, significantly suppressing transmembrane mixing. However, the introduction of hydrophilic ZrO_2_ nanoparticles did not compromise the proton conductivity of the SPEEK-based membrane, a phenomenon that is fundamentally different from the interaction between inorganic fillers, such as that resulting from incorporating SiO_2_ and TiO_2_ in the SPEEK matrix. Furthermore, testing of VRFBs showed that the hybrid membrane maintained EE of 90% over 100 charge–discharge cycles at 120 mA cm^−2^, significantly outperforming the cycling performance of the pristine SPEEK membrane. By demonstrating the applicability and performance advantages of the SPEEK–ZrO_2_ hybrid membrane in VRFBs, this study differentiates itself from previous studies focused on fuel cell applications and provides new practical insights for the development of PEMs for flow battery applications.

## 2. Materials and Methods

### 2.1. Materials

Poly(ether ether ketone) (PEEK, 450PF) powder was purchased from Victrex, Lancashire, UK. Concentrated sulfuric acid and N,N-dimethylformamide (DMF) were obtained from Xilong Science & Technology Co., Ltd. (Shantou, China). and Tianjin Fuyu Fine Chemical Co., Ltd. (Tianjin, China)., respectively. The zirconium dioxide (ZrO_2_, Z104402, Mw: 123.22) particles were purchased from Aladdin (Shanghai, China). Graphite felts (GFs) with a thickness of 4.35 mm were purchased from Wuhan Zhisheng New Energy Co., Ltd. (Wuhan, China). The Nafion 115 (N115) membrane was provided by Suzhou Sinero Technology Co., Ltd. (Suzhou, China).

### 2.2. Preparation of SPEEK and SP–Z Hybrid Membranes

SPEEK (DS = 74%) fibers were synthesized by post-sulfonation employing concentrated sulfuric acid as the sulfonating reagent. The specific DS was selected because it has been reported in the literature to possess the best physicochemical and electrochemical properties in VRFB applications [44]. PEEK polymer (2 g) was gradually added to 20 mL of concentrated sulfuric acid within 1 h at room temperature and then heated in a water bath at 55 °C for 3 h. Subsequently, the solution was poured into an ice–water mixture to form a filamentous white solid precipitate under mechanical stirring. The precipitate was filtered and repeatedly washed with massive deionized water until neutral. The obtained polymer was dried overnight in an oven at room temperature, followed by drying at 60 °C for 8 h and further drying at 100 °C for 5 h.

**Figure 1:**
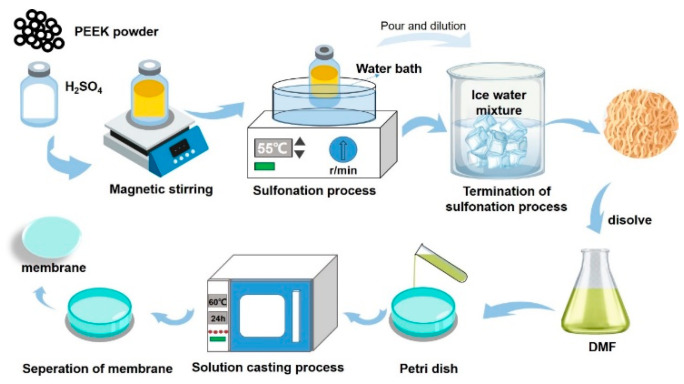
The sulfonation process and the procedures for membrane preparation.

The composite membranes were prepared by solution casting. A certain mass of SPEEK fibers was completely dissolved in DMF solvent under stirring. Various quantities of dried zirconium oxide powder were added to the DMF solvent and stirred for dispersion at 60 °C for 5 h. After ultrasonic treatment at 60 °C for one hour, the dispersion was mixed with the polymer solution and stirred overnight at room temperature. Then, the obtained mixture was sonicated at 60 °C for 1 h to disperse the nanoparticles more evenly. The mixture was then poured onto a flat petri dish and dried at 60 °C for 24 h and then at 100 °C for 12 h to evaporate the DMF solvent. Finally, the SPEEK-based membranes obtained were peeled off the glass plate to obtain the SP–Z-X membranes, where the value of X stood for the mass percentage of ZrO_2_ in the mass of SPEEK matrix. X = 0 indicated pristine SPEEK membranes without the incorporation of ZrO_2_. The sulfonation process and the procedures for membrane preparation using the solution casting method are shown in Figure 1.

### 2.3. Membrane Characterizations

#### 2.3.1. Morphology

The surface morphology of SPEEK and SP–Z composite membranes was captured by scanning electron microscopy (SEM, Zeiss 560, Carl Zeiss AG, Oberkochen, Germany). The samples were dried at 80 °C before testing and then the surface was sprayed with gold for testing.

#### 2.3.2. FTIR

The structure of the hybrid membrane was characterized using a Great 20 Fourier transform infrared spectrometer (FTIR) produced by Great 20, Zhongke Ruijie Technology Company, Tianjin, China., and different modes of the functional groups were identified. The wavenumber range was 500–4000 cm^−1^, with 32 scans and a resolution of 4 cm^−1^.

#### 2.3.3. XRD of ZrO_2_

X-ray diffraction was performed using an X’Pert Pro diffractometer with Cu Kα radiation in the 2θ range of 10–90°. Elemental analysis of zirconia was carried out using an X-ray photoelectron spectrometer (Thermo Scientific ESCALAB 250Xi, PANalytical B.V., Almelo, The Netherlands). The average grain size of zirconia was calculated using the Scherrer formula:

(1) $$d=\frac{0.9\lambda }{\beta cos(\theta )}$$

where θ is the Bragg angle of the peak, β is the full width at half maximum (FWHM), and λ of 0.15418 nm was used.

#### 2.3.4. Ion-Exchange Capacity (IEC) and DS

The IEC of the PEM was determined by acid–base titration [48,49,50]. A certain mass of dry membrane was taken and weighed with an electronic balance. After the membrane had dried, it was immersed in saturated NaCl solution for 24 h to fully exchange H^+^ in the membrane to the form of Na^+^. Subsequently, the obtained solution was titrated with 0.1 M NaOH solution until neutrality. The IEC value was calculated by the following formula:

(2) $$IEC=\frac{V_{NaOH}\times C_{NaOH}}{W_{d}}$$

where $V_{NaOH}$ is the volume of NaOH solution consumed for titration (mL) $C_{NaOH}$ is the molar concentration of NaOH solution (L^−1^), and $W_{d}$ is the mass of the dry membrane (g).

The DS of the membrane was calculated by the following formula:

(3) $$DS=\frac{M_{PEEK}\times IEC}{1000-80\times IEC}\times 100\%$$

where M_PEEK_ is the molar weight of the PEEK repeating unit (288 g mol^−1^), 1000 is the calculation coefficient, and 80 is the molar mass increased by PEEK to SPEEK (g mol^−1^).

#### 2.3.5. Water Uptake (WU) and Swelling Ratio (SR)

To carry out the water uptake test, the prepared membrane was weighed in a dry state (dried in a thermostatic oven at 80 °C overnight) to obtain the dry membrane mass. The membrane was then immersed in deionized water for 24 h, enabling it to fully absorb water. Finally, the wet membrane mass was weighed. The water uptake of the membrane was calculated according to the following formula:

(4) $$WU=\frac{W_{w}-W_{d}}{W_{d}}\times 100\%$$

For the swelling ratio test, a dry rectangular membrane sample was cut and the initial length D_d_ of the membrane sample was measured. Then, the sample was immersed in deionized water at a constant temperature for 24 h, which allowed it to fully absorb the water. After taking out the sample, the wet size D_w_ was measured and the SR calculated employing the following formula:

(5) $$SR\left(\%\right)=\frac{D_{w}-D_{d}}{D_{d}}\times 100\%$$

where D_w_ is the length of the sample in a wet state (after immersion; μm) and D_d_ is the length of the sample in a dry state (μm).

#### 2.3.6. Conductivity of the Membranes

The membranes were cut into 3 cm × 3 cm squares and placed in a proton conductivity fixture (Wuhan Chuxin Technology Co., Ltd. from Wuhan, China.). The proton conductivity was calculated by measuring the electrochemical impedance spectrum (EIS) using a two-electrode method and a GAMRY Interface 5000e (Gamry Electrochemical Instrument Company, Warminster, PA, USA) electrochemical workstation. The test frequency was 1–10^6^ Hz and the disturbance amplitude 10 mV. The specific calculation formula was as follows:

(6) $$\sigma =\frac{d}{RS}$$

where σ is the proton conductivity of the membrane (S cm^−1^), d is the distance between the two platinum electrodes, and R and S are the resistance (Ω) and cross-sectional area (cm^2^) of the measured membrane, respectively.

#### 2.3.7. Thermogravimetric Analysis

The thermal stability performance of the membranes was tested on an STA401 (Nanjing Dazhan Testing Instrument Co., Ltd., Nanjing, China), with a heating range from room temperature to 700 °C. Before the TGA measurement, the membrane sample used for the TGA test was dried in an oven at 80 °C overnight. Then, the sample was transferred directly from the oven to the TGA instrument. The temperature ramp rate was set at 10 °C min^−1^, and air was employed for the gaseous atmosphere.

#### 2.3.8. Vanadium Ion Permeability

**Figure 2:**
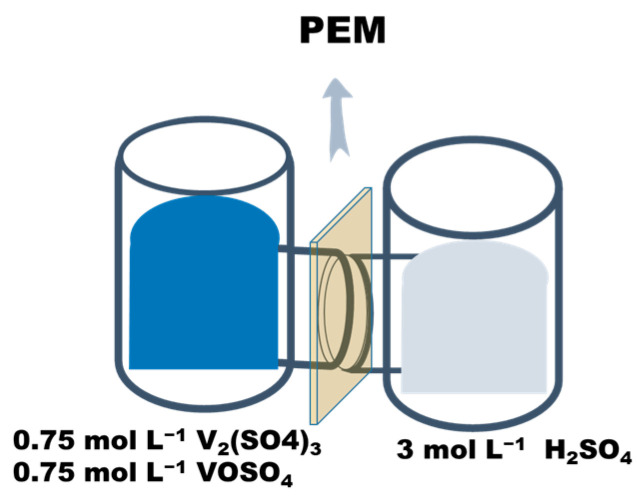
Schematic diagram of vanadium permeability test fixture.

The crossover of VO^2+^ across the membranes was measured by sandwiching the obtained proton-exchange membranes between two L-shape diffusion cells [51,52]. The test fixture is shown in Figure 2. In detail, 25 mL of mixed solution of 0.75 mol L^−1^ 1:1 mixture of V_2_(SO_4_)_3_ and VOSO_4_ in 3 mol L^−1^ H_2_SO_4_ was added to the left diffusion cell, and 25 mL of a mixed solution of 3 mol L^−1^ H_2_SO_4_ was added to the right diffusion cell. Magnetic stirring was applied to both diffusion cells to reduce concentration polarization. At certain time points, 3 mL of the solution in the right diffusion cell was taken for UV-vis spectrophotometry detection of absorbance. After the test was completed, the solution was poured back into the right diffusion cell. The permeability of VO^2+^ was calculated based on the following formula:

(7) $$V_{R}\frac{dC_{R}\left(t\right)}{dt}=\frac{A\times P}{L}\left(C_{L}-C_{R}\left(t\right)\right)$$

where V_R_ is the volume of solution in the right diffusion cell, C_R_ is the concentration of VO^2+^ on the right side of the diffusion cell, t is the time for the permeability test, A is the effective area of the PEM (1.7 cm^2^), L is the thickness of the PEM, P is the permeability of VO^2+^ through the membrane, and CL is the concentration of VO^2+^ on the left side of the diffusion cell.

Ion selectivity was defined as the ratio of the proton conductivity of the membrane to the VO^2+^ permeability, which was employed to comprehensively evaluate the performance of the obtained membranes in VRFBs:

(8) $$S=\frac{\sigma }{P}$$

where S is the ionic selectivity of the membrane, σ is the proton conductivity of the membrane, and P is the vanadium permeability.

#### 2.3.9. Chemical Stability Test

The membrane sample—mass W_0_, thickness L_0_—was dried and weighed. Then, the sample was immersed in a mixed solution consisting of 1.5 mol L^−1^ V (V) and 3 mol L^−1^ H_2_SO_4_. After immersion for 48 h, the sample was taken out and weighed after removing surface moisture with absorbent paper. The mass of the sample after immersion and moisture removal was W_1_ and the thickness L_1_. The chemical stability of the membrane was evaluated according to the changes in weight and thickness of the samples. The relevant calculation formula is as follows:

(9) $$d\;Weight\left(\%\right)=\frac{W_{0}-W_{1}}{W_{0}}\times 100\%$$

(10) $$d\;Length\left(\%\right)=\frac{L_{0}-L_{1}}{L_{0}}\times 100\%$$

#### 2.3.10. Single-Cell Tests

The single-cell performance of VRFBs assembled with SPEEK and SPEEK–ZrO_2_ hybrid membranes was tested on single cells provided by Wuhan Zhisheng New Energy Co., Ltd. (Wuhan, China). A comprehensive characterization and examination of the practical electrochemical performance of a VRFB single cell within a voltage range of 0.7 to 1.6 volts was carried out. The electrolytes were formulated using 1.5 M vanadyl sulfate trihydrate (VOSO_4_·3H_2_O, Haizhongtian Chemical Company, Shenyang, China) and 3M sulfuric acid (H_2_SO_4_, 98%). During preparation, the electrolysis process yielded an anolyte containing 1.5 M V(III) and 3.0 M H_2_SO_4_ (40 mL) and a catholyte containing 1.5 M V(IV) and 3.0 M H_2_SO_4_ (40 mL). A series of charge–discharge tests were conducted at different current densities between 40 mA cm^−2^ and 200 mA cm^−2^ to assess the CE, voltage efficiency (VE), and EE of VRFBs equipped with different PEMs. The calculation formulas for CE, VE, and EE of a VRFB were as follows:

(11) $$CE\left(\%\right)=\frac{\int I_{d}dt}{\int I_{c}dt}\times 100$$

(12) $$EE\left(\%\right)=\frac{\int V_{d}I_{d}dt}{\int {V_{c}I}_{c}dt}\times 100$$

(13) $$VE\left(\%\right)=\frac{EE}{CE}\times 100$$

where I_d_ represents the discharging current, I_c_ represents the charging current, t represents time, V_d_ represents the discharging voltage, and V_c_ represents the charging voltage.

The self-discharging was then evaluated by measuring the open-circuit potential (OCP) of a VRFB mounting the selected membrane as a function of time starting from a fully charged state obtained as described above (i.e., the VRFB was charged until a cutoff voltage of 1.65 V was reached) and until a cutoff value of 0.8 V was achieved.

## 3. Results and Discussion

### 3.1. Morphology of Membranes

**Figure 3:**
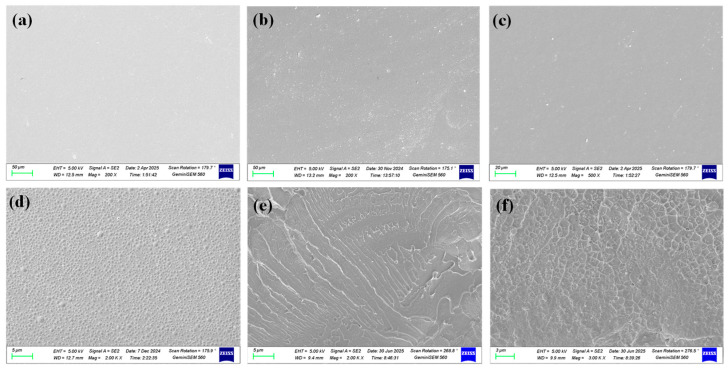
Surface morphology of SPEEK and SP–Z-X composite membranes: (**a**) SPEEK; (**b**) SP–Z-2.5; (**c**) SP–Z-5; (**d**) SP–Z-7.5. Cross section morphology of SPEEK and SP–Z-X composite membranes: (**e**) SPEEK; (**f**) SP–Z-5.

The obtained pristine SPEEK membrane and SP–Z composite membranes were synthesized through solution casting. By SEM characterization, it can be concluded that both the pristine SPEEK membrane and SP–Z composite membranes exhibited uniform and dense surface morphology, as shown in Figure 3a,b, without microscopic pores and other structural defects. Even under high-resolution SEM, both the pristine SPEEK membrane and SP–Z composite membranes show good morphology, as shown in Figure 3c,d. It can be concluded that zirconia nanoparticles are uniformly dispersed in the SPEEK polymer matrix.

### 3.2. Fourier Transform Infrared (FTIR) Spectroscopy

**Figure 4:**
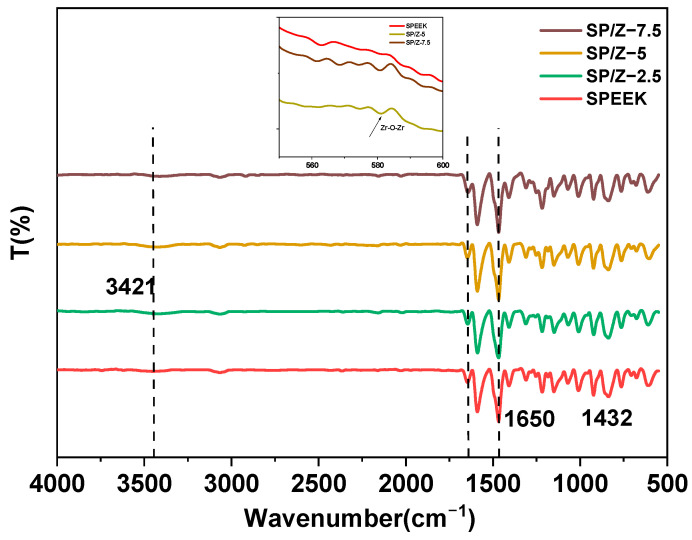
Infrared spectra of SPEEK and SP–Z-X hybrid membranes.

Figure 4 shows the infrared spectra of the pristine SPEEK membrane and SP–Z hybrid membranes. As can be seen, the shapes of the infrared spectra of the pristine SPEEK membrane and the composite membranes are almost identical. The SPEEK membrane exhibits a characteristic broad peak of the hydroxyl groups in the sulfonic acid group at 3421 cm^−1^. The absorption peak of the -SO_3_H groups in the SPEEK membrane at 1432 cm^−1^ is due to the asymmetric stretching vibration of O=S=O, while the absorption peak at 1076 cm^−1^ is caused by the symmetric stretching vibration of O=S=O. The stretching vibrations of S-O and S=O correspond to 709 cm^−1^ and 1161 cm^−1^. The intensity of these four characteristic absorption peaks indicates the content of -SO_3_H groups in the SPEEK membranes. The higher intensity of the characteristic absorption peak indicates more -SO_3_H groups and the higher DS of the SPEEK membranes. As a result of the addition of ZrO_2_ nanoparticles, a distinct absorption peak for the symmetric stretching vibration of Zr-O-Zr can be observed at 580 cm^−1^, indicating that zirconia has been successfully incorporated into the composite membranes. The infrared test results were consistent with those described in the literature [53,54,55]. Slight deviations in O=S=O characteristic peaks were detected in the hybrid membranes in comparison to the pristine SPEEK membrane, demonstrating the appearance of intermolecular hydrogen bonds between the sulfonate groups of SPEEK and the zirconium dioxide nanoparticles.

### 3.3. XRD of ZrO_2_

**Figure 5:**
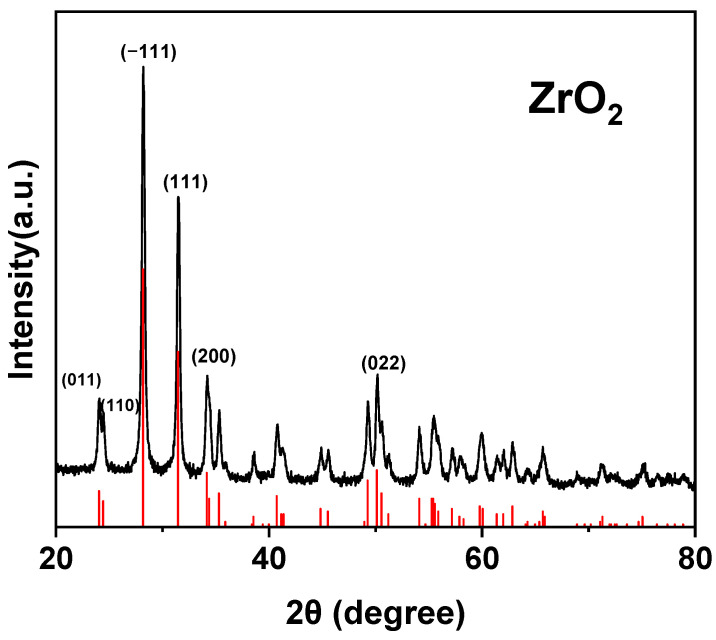
XRD pattern of zirconia nanoparticles.

Figure 5 shows the X-ray diffraction pattern of zirconia nanoparticles, which is consistent with the standard card. The results indicate that the strongest peak appears at 2θ = 28.20960°, corresponding to the (−111) plane of the monoclinic phase of zirconia, with a full width at half maximum (FWHM) of 0.318°. The results demonstrate that the sample is primarily composed of the monoclinic phase of zirconia, with good crystallinity and high purity. According to the Scherrer formula, the average grain size of the nanoparticles was calculated to be approximately 27 nm.

### 3.4. Characterization of the Physicochemical Features of Membranes

Physicochemical properties of Nafion 115, the pristine SPEEK membrane, and SP–Z composite membranes are summarized in Table 1. As shown in Table 1, the introduction of ZrO_2_ increased the hydrophilicity of the hybrid membranes. The swelling ratio (SR) and water uptake (WU) of the hybrid membranes increased within a certain range with the increase in nano-oxide content, and increased water absorbed in the membranes was beneficial for improving proton conductivity. SP–Z-5 demonstrated the highest water uptake (71.1%) and swelling ratio (25.33%). The swelling ratio of the pristine SPEEK membrane was 19.23%, while that of composite membranes ranged from 23.51% to 25.33%. The SR and proton conductivity of SP–Z-5 were the highest, which may have been related to its high water uptake.

Ion-exchange capacity (IEC) is a pivotal index for weighing the capability of PEMs. The IEC of the pristine SPEEK membrane was 2.12 mmol g^−1^, while the IEC of the hybrid membranes was between 1.63 and 2.00 mmol g^−1^. The addition of ZrO_2_ diluted the concentration of -SO_3_H groups in the composite membranes, resulting in a reduction in the IEC of the membrane with an enhancement in the nano-oxide content. Proton conductivity is another critical parameter for evaluating the performance of proton-exchange membranes.

As can be seen in Table 1, the proton conductivity of the pristine SPEEK was 0.096 S cm^−1^, while that of the hybrid membranes ranged from 0.111 to 0.240 S cm^−1^. This reveals that incorporation of zirconium oxide enhances the proton conductivity of the hybrid membranes. Among these hybrid membranes, the SP–Z-5 membrane exhibited the highest proton conductivity. This phenomenon may be related to its high water uptake, which is conducive to accelerating the migration rate of protons. The proton conductivity of the SP–Z-10 hybrid membrane decreased, which may have been due to aggregation of the inorganic phase wherein the content of inorganic nanoparticles rose to a certain extent, leading to limited hydrophobic and hydrophilic phase separation with the SPEEK matrix and resulting in microscopic defects and a decrease in membrane performance.

### 3.5. Thermal Stability Analysis of the Membranes

Thermal stability is one of the key performance indicators of PEMs. To simulate the oxygen-containing environment in actual material applications and evaluate its long-term stability, this study selected an air atmosphere for TGA testing. Studies have reported that compared with nitrogen atmosphere, oxygen in an air environment will undergo an oxidation reaction with SPEEK, causing the decomposition temperature of the membrane to be slightly lower than that in a nitrogen environment. It may also increase the weight loss stage and rate and reduce the amount of residual carbon [50,56].

**Figure 6:**
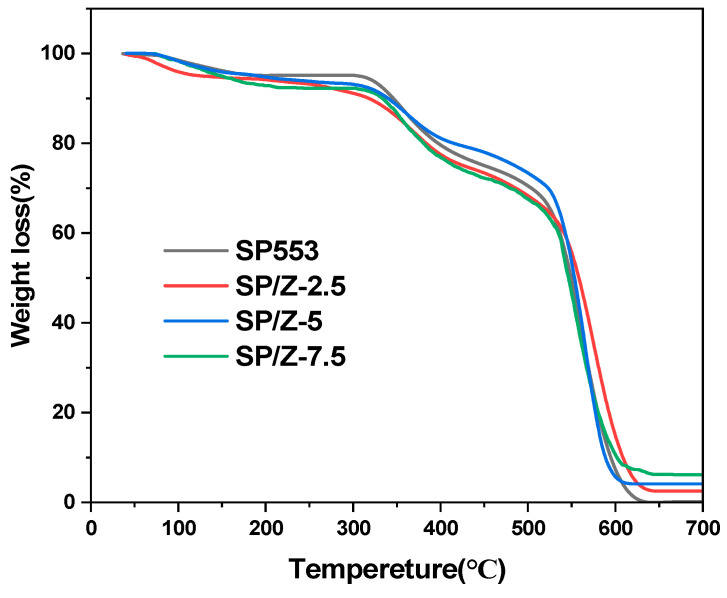
TGA curves of SPEEK and SP–Z-X hybrid membranes.

Figure 6 illustrates the thermal mass loss curves of the pristine SPEEK and hybrid membranes. As can be seen, the temperature range of 25–150 °C can be assigned to the evaporation of water on the PEM surface and in the PEM. The weight loss starting from 350 °C corresponds to the degradation of sulfonate groups in the SPEEK matrix. The temperature at which the main chain of SPEEK matrix begins to degrade due to oxidative decomposition, starting from approximately 500 °C, is similar to other reported values [57,58]. Within the temperature range of 20–140 °C, the thermal stability of all membranes is comparable, indicating that introducing zirconia nanofillers will not have a negative impact on the thermal stability of the SPEEK matrix. When the temperature is above 160 °C, the addition of ZrO_2_ nanofiller reduces the thermal stability of the composite membrane to a certain extent. It can be seen from the TGA curve that SPEEK begins to decompose at 160 °C, but SPEEK and SP–Z composite membranes are fully capable of stable operation at normal operating temperatures (10–40 °C) of VRFBs.

### 3.6. Vanadium Ion Permeability of Membranes

**Figure 7:**
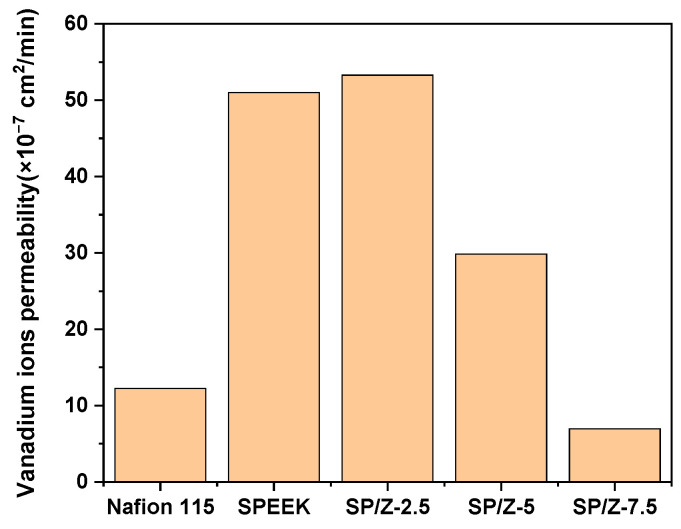
Vanadium species crossover results of Nafion 115, SPEEK, and SP–Z-X membranes.

The vanadium species crossover of the PEM should be reduced as much as possible for VRFBs, which significantly affects the comprehensive output performance of the battery. The variation curves of the transmembrane transport concentration and permeability of VO^2+^ of Nafion 115, SPEEK, and their composite membranes over time are shown in Figure S1 and Figure 7. The results indicate that the permeability of VO^2+^ across Nafion 115 is 12.25 × 10^−7^ cm^2^/min, and its large thickness hinders the migration of vanadium ions in comparison to that of Nafion 212 (55.8 × 10^−7^ cm^2^/min), as reported in the literature [57]. The vanadium ion crossover of the pristine SPEEK membrane is 51.01 × 10^−7^ cm^2^/min. After the addition of inorganic nanoparticles, the presence of nanoparticles suppresses the migration of vanadium active species, making the transmembrane transport channel of vanadium ions narrower, thereby reducing the permeability of VO^2+^. SP–Z-7.5 possesses the strongest capability to inhibit the crossover of vanadium species, and the vanadium ion permeability is only 6.97 × 10^−7^ cm^2^/min. The obtained results are consistent with descriptions in other literature [59,60,61,62].

**Figure 8:**
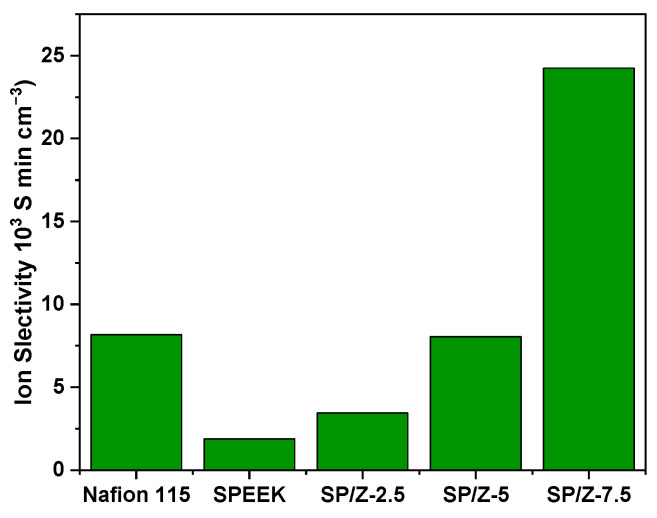
Ion selectivity of Nafion 115, SPEEK, and SP−Z-X membranes.

Taking into account the proton conductivity and the ability to inhibit the migration of vanadium species, SP–Z-7.5 is considered the membrane with the best performance for VRFBs. By comparing the ionic selectivity of membranes, the performance of the membrane can be evaluated more accurately. The ionic selectivity of the membrane is defined as the ratio between proton conductivity and vanadium ion permeability. Figure 8 demonstrates the ionic selectivity comparison of Nafion 115, SPEEK, and SP–Z-X composite membranes. It can be seen in the figure that the incorporation of ZrO_2_ nanoparticles effectively enhances the selectivity by inhibiting the crossover of vanadium species. The ion selectivity enhances with the addition of zirconium oxide nanoparticles, and the selectivity of the SP–Z-7.5 membrane reaches its maximum value (24.25 × 10^3^ S min cm^−3^). This increase in selectivity is due to the fact that the inorganic nanoparticles also synergistically promote the proton conductivity of the membrane and form stable proton transport channels. SP–Z-5 (8.0 × 10^3^ S min cm^−3^) has comparable ion selectivity to Nafion 115 (8.16 × 10^3^ S min cm^−3^). Therefore, the SP–Z-7.5 membrane is considered the membrane with the best ion selectivity.

### 3.7. Chemical Stability

**Figure 9:**
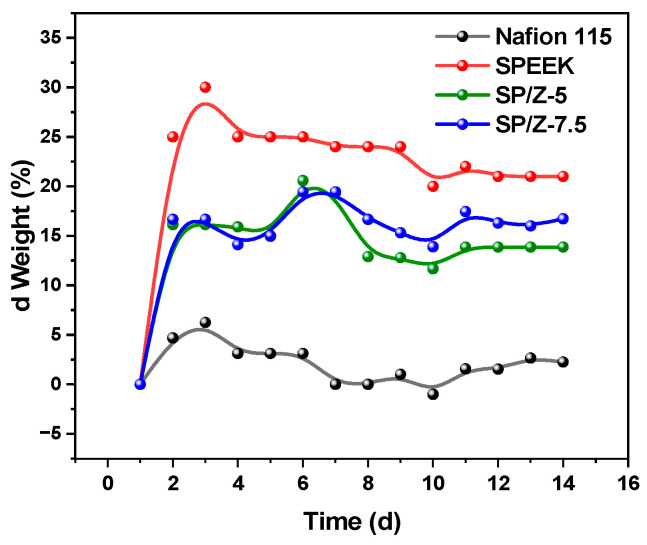
Relative variation in weight of Nafion 115, Pristine SPEEK, and SP–Z-X membranes as a function of immersion time in a solution consisting of 1.5 M VO^2+^ in 3M H_2_SO_4_.

**Figure 10:**
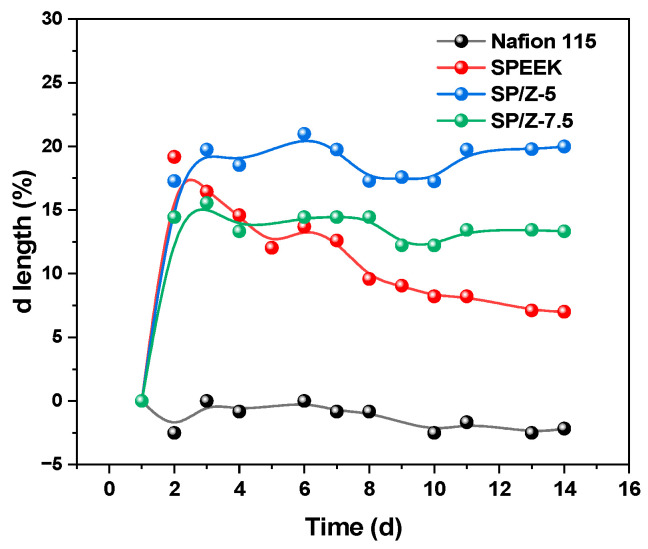
Relative variation in thickness of Nafion 115, Pristine SPEEK, and SP–Z-X membranes as a function of immersion time in a solution consisting of 1.5 M VO^2+^ in 3M H_2_SO_4_.

The oxidation stability of Nafion 115, SPEEK and their hybrid membranes was measured and compared. The membranes were soaked in a mixed solution of 1.5 M V (V) and 3 M H_2_SO_4_ for two weeks, and the thickness and weight variations of the membranes were recorded. The SPEEK membrane exhibited the largest weight change, while the Nafion 115 membrane demonstrated the lowest weight change and thickness change, as shown in Figure 9 and Figure 10. The results indicated that Nafion 115 had the best chemical and dimensional stability and the chemical stability of the SPEEK membrane was still some way off Nafion 115. However, after the addition of zirconium oxide, chemical stability was promoted to a certain extent. By introducing inorganic nanoparticles into the SPEEK matrix, the chemical stability of the membrane was enhanced, and the oxidative degradation of the membrane in VRFBs was inhibited to a certain extent. This is because zirconium oxide forms hydrogen bonds with the -SO_3_H groups in SPEEK, thereby enhancing the chemical stability of the membrane. The test results demonstrated that the mass loss of all membranes was very small and that they were suitable to be employed for long-term VRFB operation.

### 3.8. VRFB Single-Cell Performance

**Figure 11:**
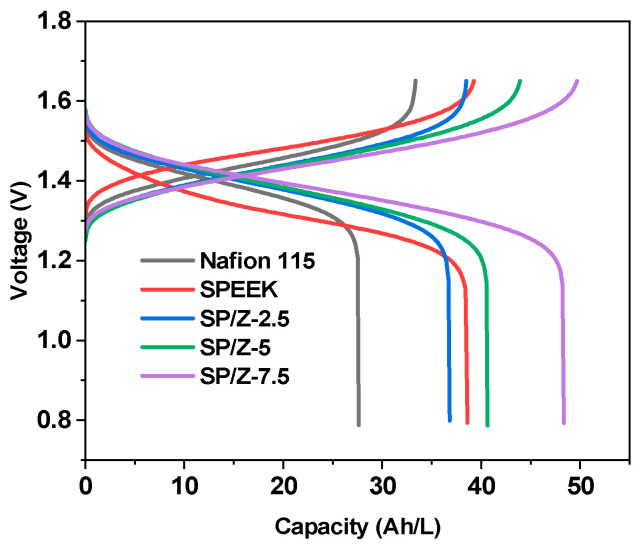
Charge–discharge curves of Nafion 115, SPEEK, and SP–Z-X membranes at 40 mA/cm^2^.

Figure 11 illustrates typical charge–discharge curves of VRFB single cells using Nafion 115, SPEEK, and SP–Z-X composite membranes at 40 mA/cm^2^. Typically, higher surface resistance will lead to a larger IR drop [63]. The results indicate that SPEEK exhibits the highest initial charging voltage and the lowest initial discharging voltage. This is attributed to its lowest proton conductivity, resulting in the most severe ohmic losses and consequently the highest overpotential. In contrast, the SPEEK membrane shows an improvement in capacity (36 Ah/L). However, its higher voltage polarization also reveals an inherent drawback of insufficient proton conduction performance. Since the conductivity of Nafion 115 is only slightly higher than that of the SPEEK membrane and Nafion 115 has the greatest thickness—about 1.5–2.5 times that of other SPEEK membranes—its charge–discharge behavior is similar to SPEEK. Nevertheless, its charge–discharge capacity and electrolyte utilization are the lowest among all membranes, with a discharge capacity of about 28 Ah/L. Notably, the SP–Z composite membranes demonstrate significant advantages in terms of capacity. When the zirconia content is 2.5 wt%, the introduction of zirconia enhances the hydrophilicity of the membrane material, thereby increasing both proton conductivity and vanadium permeability compared with SPEEK. The increased proton conductivity leads to a lower overpotential than SPEEK, but the higher vanadium permeability results in a lower discharge capacity. Because the physical barrier mechanism is not yet evident at this limited ZrO_2_ loading, the charge–discharge capacity of the SP–Z-2.5 composite membrane is slightly reduced relative to pure SPEEK. The ZrO_2_-doped SPEEK composite membranes (SP–Z) achieve a breakthrough in performance balance: the SP–Z-5 membrane attains a superior discharge capacity (48 Ah L^−1^), attributed to the optimized modification of ionic channels by nanoparticles, which enhances vanadium-blocking ability. Although its overpotential is higher than that of Nafion 115, it is significantly lower than that of SP–Z-7.5. The results demonstrate that moderate ZrO_2_ doping (5 wt%) can synergistically improve the selectivity and electrochemical stability of SPEEK membranes, offering a new strategy for designing high-capacity, low-cost proton-exchange membranes.

**Figure 12:**
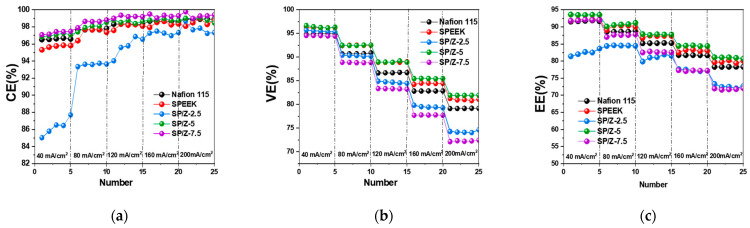
Cycling performance of VRFB single cells consisting of Nafion 115 and SP–Z-X membranes at various current densities: (**a**) CE; (**b**) VE; (**c**) EE.

Figure 12 shows the performance of VRFB single cells (including CE, VE and EE) at charge–discharge current densities of 40–200 mA/cm^2^ for Nafion 115, SPEEK, and SP–Z-X composite membranes. Generally, the high permeability of vanadium species across the PEM during VRFB operation results in the loss of electrochemical energy. When the current density increases, CE tends to rise, which is mainly because the shorter vanadium ion penetration time at high current density effectively suppresses the capacity decay originating from self-discharge phenomenon.

It can be seen that under 40 mA/cm^2^, the CEs of the VRFB single cell using the SP–Z-7.5 composite membrane is 97.48%, which is higher than the CE of the VRFB single cell using Nafion 115 of 96.62%. This fully confirms the lower vanadium species crossover of the SP–Z-7.5 hybrid membrane (6.97 × 10^−7^ cm^2^/min vs. Nafion 12.25 × 10^−7^ cm^2^/min, see Table 1). Within the entire current density ranges employed, the SP–Z-7.5 composite membrane exhibited the highest CE among all tested samples. The CE results of the membrane in the cycling test of VRFB single cells are basically consistent with the results in the vanadium ion permeation test.

The VE of different membranes in VRFBs is usually related to the magnitude of the area resistance. The lower the area resistance, the higher the VE, which is related to the lower ohmic losses. Therefore, as the current density grows, the ohmic loss is gradually enhanced, while the VE tends to decrease. At 200 mA/cm^2^, which was the highest current density tested, the VE of VRFB based on Nafion 115, SPEEK, and SP–Z-5 composite membranes was 94.99%, 96.22%, and 96.65%, respectively.

**Figure 13:**
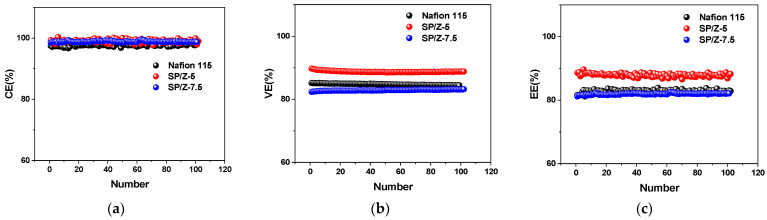
The cycling performance of VRFB single cells consisting of Nafion 115, SP–Z-5, and SP–Z-7.5 membranes at 120 mA cm^−2^: (**a**) CE; (**b**) VE; (**c**) EE.

Figure 13 shows the long-term cycling stability results of Nafion 115, SP–Z-5, and SP–Z-7.5 composite membranes. As can be seen, during the 100 cycles of charge–discharge testing at a current density of 120 mA cm^−2^, no significant cell performance degradation was observed, indicating that these membranes are all suitable for long-term operation of VRFBs. The SP–Z-5 composite membrane has the highest energy efficiency (EE), which is stable at around 90%. SP–Z-7.5 shows an EE comparable to that of the Nafion 115 membrane, both of which are stable at around 80%.

For an electrochemical energy storage system, EE is the most critical performance evaluation indicator. The EE is the product of CE and VE. With increased current density, EE decreases, which indicates that VE plays a leading role in determining EE. At the highest employed current density of 200 mA/cm^2^, compared to the EE of 91.85% for Nafion 115, the EE of VRFB using SPEEK and SP–Z-5 composite membranes was 92.21% and 93.59%, respectively. The entire battery cycling test lasted more than 150 h, and no significant decrease in battery performance was observed during the testing process, which indirectly proves that the prepared composite membrane has relatively good operating stability. In summary, compared to Nafion 115, the CE, VE, and EE of the SP–Z-5 composite membrane were significantly improved in the entire current density ranges tested. In particular, its superior chemical stability and low expense makes it a promising alternative candidate to Nafion membranes in VRFB systems.

**Figure 14:**
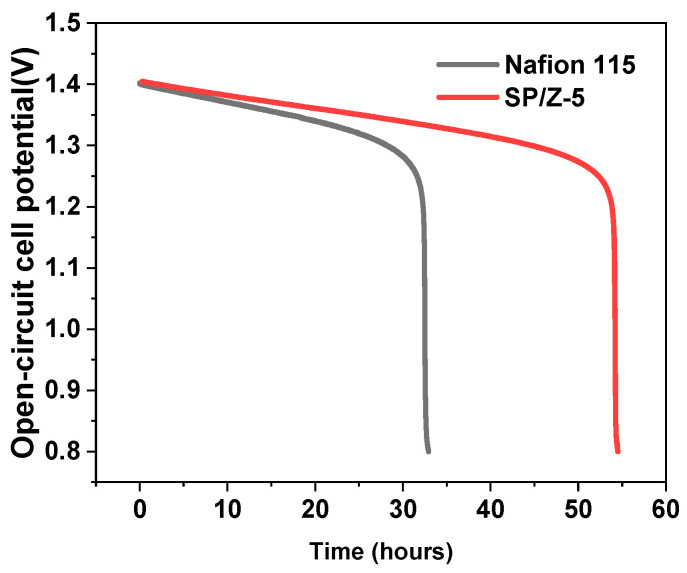
OCP curves of VRFBs equipped with Nafion 115 membrane and SP–Z-5 membrane.

Figure 14 shows the OCP curves of VRFBs assembled from Nafion 115 and SP–Z-5 hybrid membranes at 50% state of charge (SOC). The images show that the OCP of both VRFBs decreases over time due to cross-permeation of vanadium species, which reduces the concentrations of V (II) and V (V) ions in the anolyte and catholyte. The rate of decrease becomes more rapid when the OCP reaches 1.25 V, quickly reaching a cutoff value of 0.8 V. For Nafion 115 and SP–Z-5 membranes, the time to reach 0.8 V from a SOC = 50% is 33 h and 55 h, respectively. The self-discharge time of the SP–Z-5 membrane is 1.67 times that of the Nafion 115 membrane. This further demonstrates that the use of the SP–Z-5 membrane significantly reduces the in situ vanadium ion permeation, a finding inconsistent with the vanadium permeation experimental results (see Figure 5). This indicates the ex situ permeability test by UV-visible spectra may not reflect the real operational performance of the PEM in terms of self-discharging phenomenon. This is because the compression force and ratio of the membrane, the impact force of the electrolyte pushed by the pump on the membrane, and the electric field may influence permeation through the membrane significantly. Clearly, the SP–Z-5 membrane exhibits a longer charge retention capability than Nafion 115, thereby reducing the self-discharge of the single-cell VRFB.

## 4. Conclusions

In this study, a sequence of inorganic–-organic composite membranes were synthesized by solution casting with SPEEK as the matrix. The introduction of hydrophilic ZrO_2_ nanoparticles into the matrix hindered the transmembrane transport of vanadium species and lowered the vanadium crossover through the PEM. The hydrophilicity of ZrO_2_ nanoparticles further improved the WU of the hybrid membranes, thereby promoting the proton conductivity of the membranes. The results indicate that the proton conductivity of all hybrid membranes (0.111–0.240 hS cm^−1^) was higher than that of Nafion 115 (0.1 S cm^−1^), among which the WU (71.1%) and proton conductivity (0.240 S cm^−1^) of SP–Z-5 were the highest. Among all the composite membranes, SP–Z-5 had the best comprehensive output performance, at 200 mA cm^−2^ (CE: 99.01%, VE: 81.95%, EE: 81.11%), and the relevant performance indicators were higher than those of Nafion 115.

Through the characterization and analysis in this study, the reasons for the high and stable VRFB performance of SP–Z-5 hybrid membrane include the following. Firstly, the addition of hydrophilic zirconia nanoparticles increases the WU of the composite membrane, thereby increasing the proton conductivity of the hybrid membranes and enhancing the VE of the VRFB consisting of hybrid membranes. The presence of inorganic nanoparticles hinders the transmembrane transport of vanadium species and reduces the vanadium species crossover, thereby promoting the ion selectivity and CE of the PEM and thus exhibiting the best VRFB performance. As a result of the establishment of hydrogen bonds between hydrophilic zirconia and -SO_3_H groups in the SPEEK matrix, the chemical stability of the hybrid membranes and the operational stability of the battery system assembled with hybrid membranes are improved. Therefore, the SP–Z-5 hybrid membrane has excellent performance and can be considered a low-cost alternative membrane for VRFB applications. Future studies will focus on the dissolution risk of ZrO_2_ during long-term charge–discharge cycling. We plan to extend the cycling tests (more than 100 charge–discharge cycles) and combine inductively coupled plasma mass spectrometry (ICP-MS) to analyze the content of Zr elements in the electrolyte, thereby evaluating the interfacial stability of the hybrid membrane. At the same time, we will explore strategies to enhance the interactions between ZrO_2_ and the SPEEK matrix to further inhibit the dissolution of nanoparticles and improve the long-term performance of the membrane.

## Figures and Tables

**Table 1:** Physicochemical features of Nafion 115, SPEEK, and SP–Z composite membranes.

Samples	Thickness (μm)	Water Uptake (%)	Swelling Ratio (%)	IEC (mmol g^−1^)	Proton Conductivity (S cm^−1^)
Nafion 115	128	22.53	13.46	0.89	0.1
SPEEK	70	58.97	19.23	2.12	0.096
SP–Z-2.5	65	59.26	24.13	2.00	0.183
SP–Z-5	70	71.1	25.33	1.78	0.240
SP–Z-7.5	60	69.27	23.51	1.68	0.169
SP–Z-10	55	53.26	20	1.63	0.111

## Data Availability

Data are contained within the article and Supplementary Materials.

## Supplementary Materials

The following supporting information can be downloaded at https://www.mdpi.com/article/10.3390/polym17172287/s1. Figure S1: The concentration of VO^2+^ vs. time for the SPEEK/Z-X composite membranes; Table S1: Physicochemical features: swelling ratio (z direction) of Nafion 115, SPEEK, and SP–Z Composite Membranes.
