# Supplementary material for: Sulfonated Poly(ether ether ketone)–Zirconia Organic–Inorganic Hybrid Membranes with Enhanced Ion Selectivity and Hydrophilicity for Vanadium Redox Flow Batteries
Source: Polymers (Basel). 2025 Aug 23;17(17):2287. doi: 10.3390/polym17172287 (PMC12430635; doi:10.3390/polym17172287)
Supplement: Supplementary file 1 [file polymers-17-02287-s001.zip › polymers-3801084-supplementary.pdf]

polymers

Article

Sulfonated Poly(ether ether ketone)/Zirconia Organic-Inorganic Hybrid Membranes with Enhanced Ion-Selectivity and Hydrophilicity for Vanadium Redox Flow Batteries

Xiang Li [1], Tengling Ye [1], Wenfei Liu [2], Ge Meng [3], Wenxin Guo [4], Sergey A. Grigoriev [5,6], Dongqing He [7,*] and Chuanyu Sun [3,8,*]

[1] Department of Applied Chemistry, School of Chemistry and Chemical Engineering, Harbin Institute of Technology, Harbin 150001, China
[2] Yantai Research Institute, Harbin Engineering University, Yantai 264003, China
[3] School of Electrical Engineering and Automation, Harbin Institute of Technology, Harbin, 150001, China
[4] Department of Proving Ground, FAW-Volkswagen Automotive Company Limited, Changchun 130000, Jilin, P.R. China
[5] National Research University "Moscow Power Engineering Institute", 14, Krasnokazarmennaya St., Moscow, 111250, Russia
[6] National Research Centre "Kurchatov Institute", 1, Akademika Kurchatova Sq., Moscow, 123182, Russia
[7] Institute of Advanced Technology, Heilongjiang Academy of Sciences, Harbin 150020, China
[8] Suzhou Research Institute, Harbin Institute of Technology, Suzhou, 215104, China
* Correspondence: dqhe2015@163.com; chuanyu.sun@hit.edu.cn

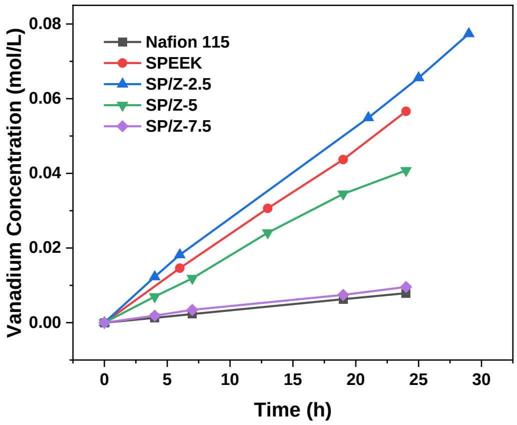

Figure S1. The concentration of VO^{2+} vs. time for the SPEEK/Z-X composite membranes.

Table S1. Physicochemical Features Swelling Ratio(z direction) of Nafion 115, SPEEK, and SP/Z Composite Membranes.

**Samples**	**Swelling Ratio along z direction (%)**
Nafion 115	21.09
SPEEK	34.55
SP/Z-2.5	18.00
SP/Z-5	16.18
SP/Z-7.5	18.188
SP/Z-10	11.118
